# Steroid-insensitive tobacco smoke-induced lung inflammation models in the mouse

**DOI:** 10.1186/1476-9255-10-S1-P31

**Published:** 2013-08-14

**Authors:** V Russell, D Spicer, P Woodman, A Connolly, A Young

**Affiliations:** 1 Argenta Discovery, Spire Green, Harlow, Essex, UK

## Background

The effects of oral and inhaled steroids were investigated in murine models of lung inflammation induced by 4 or 11 days of exposure to tobacco smoke (TS). The effects were compared to other anti-inflammatory compounds.

## Methods

Female mice were exposed daily to TS for 4 or 11 days. Control animals were exposed to air. Mice were killed 24hrs after the last TS-exposure, lungs lavaged and prepared for histological assessment. Steroids were given orally (dexamethasone (DEX) 300ug/kg; budesonide (BUD) 10mg/kg, 1hr prior to, and 6hrs after each TS exposure) or intra-nasally (fluticasone proprionate (FP), DEX , BUD, beclomethasone, mometasone and ciclesonide dosed at 100-500ug/kg 1hr prior to each TS-exposure). Steroid efficacy was also investigated in a mouse LPS lung inflammation model.

## Results

TS-exposure induced a cellular infiltration into the lungs which was reproducible across more than 50 studies. The main cell types in the lavage were macrophages and neutrophils. Histopathological assessment showed a progressive increase in pathology from 4 days of exposure (predominantly vasculitis, alveolar congestion and epithelial hyperplasia) to 11 days of exposure (bronchiolitis, pneumonitis, bronchiolar degeneration, bronchial wall remodelling). Steroids dosed via the oral and intra-nasal routes failed to inhibit this inflammation in both models (maximal inhibition of TS-induced inflammation 14%, all p>0.05 compared to controls), although they caused body weight loss, confirming systemic availability and activity. The doses of steroids used in the TS studies all showed significant efficacy in a mouse LPS-model (all >65% inhibition, p<0.05), confirming that the TS-exposure model was refractory to steroids. The TS-induced inflammation was reduced by PDE4 inhibitors such as Roflumilast when given either i.n. (total cells 50%, neutrophils 60%, both p<0.05) or orally (total cells 50%, neutrophils 66%, both p<0.05). Inhibitors of p38 MAP kinase were effective when given orally (BIRB796: total cells 42%, neutrophils 55% inhibition, both p<0.05) or i.n. (PF03715455: total cells 50%, neutrophils 61% inhibition, both p<0.05). These inhibitory effects were confirmed in multiple studies where steroids continued to lack efficacy.

## Conclusions

TS-exposure for up to 11 days induced lung inflammation. The inflammatory response was not affected by doses of steroids which were highly efficacious in a mouse LPS model. The steroid insensitive effects were robust and reproducible across multiple studies. The TS-induced inflammation was inhibited by a clinically used PDE4 inhibitor (Roflumilast) and p38 inhibitors such as PF03715455, which is currently in phase I clinical development.

